# Trace elements as tumor biomarkers and prognostic factors in breast cancer: a study through energy dispersive x-ray fluorescence

**DOI:** 10.1186/1756-0500-5-194

**Published:** 2012-07-06

**Authors:** Marina P Silva, Danilo F Soave, Alfredo Ribeiro-Silva, Martin E Poletti

**Affiliations:** 1 Departamento de Física, Universidade de São Paulo, FFCLRP, Av. dos Bandeirantes n. 3900, 14040-901, Ribeirão Preto, SP, Brazil; 2 Departamento de Patologia, FMRP/USP, Av. dos Bandeirantes n. 3900, 14049-900, Ribeirão Preto, SP, Brazil

## Abstract

**Background:**

The application and better understanding of traditional and new breast tumor biomarkers and prognostic factors are increasing due to the fact that they are able to identify individuals at high risk of breast cancer, who may benefit from preventive interventions. Also, biomarkers can make possible for physicians to design an individualized treatment for each patient. Previous studies showed that trace elements (TEs) determined by X-Ray Fluorescence (XRF) techniques are found in significantly higher concentrations in neoplastic breast tissues (malignant and benign) when compared with normal tissues. The aim of this work was to evaluate the potential of TEs, determined by the use of the Energy Dispersive X-Ray Fluorescence (EDXRF) technique, as biomarkers and prognostic factors in breast cancer.

**Methods:**

By using EDXRF, we determined Ca, Fe, Cu, and Zn trace elements concentrations in 106 samples of normal and breast cancer tissues. Cut-off values for each TE were determined through Receiver Operating Characteristic (ROC) analysis from the TEs distributions. These values were used to set the positive or negative expression. This expression was subsequently correlated with clinical prognostic factors through Fisher’s exact test and chi-square test. Kaplan Meier survival curves were also evaluated to assess the effect of the expression of TEs in the overall patient survival.

**Results:**

Concentrations of TEs are higher in neoplastic tissues (malignant and benign) when compared with normal tissues. Results from ROC analysis showed that TEs can be considered a tumor biomarker because, after establishing a cut-off value, it was possible to classify different tissues as normal or neoplastic, as well as different types of cancer.

The expression of TEs was found statistically correlated with age and menstrual status. The survival curves estimated by the Kaplan-Meier method showed that patients with positive expression for Cu presented a poor overall survival (*p* < 0.001).

**Conclusions:**

This study suggests that TEs expression has a great potential of application as a tumor biomarker, once it was revealed to be an effective tool to distinguish different types of breast tissues and to identify the difference between malignant and benign tumors. The expressions of all TEs were found statistically correlated with well-known prognostic factors for breast cancer. The element copper also showed statistical correlation with overall survival.

## Background

Today, diagnosis and therapeutic approach for breast cancer is based on predictive and prognostic factors which are well-established for this disease. Prognostic factors such as tumor size, lymph nodal status, TNM staging information, histological grade and type, mitotic figure counts and hormone receptor status have proven to be of prognostic importance and useful in clinical patient management [1]. Other prognostic factors have been extensively studied biologically and clinically, but their importance remains to be validated in statistically robust studies, including c-erbB-2 (Her2-neu) [2], VEGF [3], p53 expression [4], among others [1]. The combination of two or more parameters in order to define the prognosis of the disease can be of considerable importance, since it makes it possible to define the risk and to indicate the potential value or not of a certain treatment [5].

Several prognostic and predictive factors can act as tumor biomarkers, depending on the given treatment. Biomarkers are any type of measurable element which is able to demonstrate the presence of malignancy or malignant potential, or to predict the behaviour of the tumor, the prognosis or the treatment response [6]. A better understanding and application of traditional tumor biomarkers and the identification of new markers is essential since they improve the patients quality of life by sparing them from going under toxic treatments that are unlikely to benefit them, and also by making it possible to establish an appropriate individualized treatment for each type of tumor, avoiding unnecessary treatment [5,6].

In recent years, the analysis of trace elements in human tissues has gained great interest due to the role that these elements play in biochemical and physiological processes. Although trace elements constitute a minor part of living tissues, they are important for vital processes [7]. Some metals, usually present in proteins, enzymes and cellular membranes, are essential for the normal physiological function [8-10]. However, when in abnormal expression, they seem to contribute in several pathological processes, including tumor growth, invasion and metastasis [11-13]. Individually, these elements seem to contribute to various pathological processes, although all the roles of these metals in carcinogenesis are still unknown [14-20]. Previous publications of our group highlighted the study of some elements, such as calcium, iron, copper and zinc, by determining the concentrations of these elements in breast tissue by X-Ray Fluorescence (XRF) techniques [7,21-23]. These studies showed, in agreement with others [7,21,22,24-30], that these trace elements are found in significantly higher concentrations in neoplastic breast tissues (malignant and benign) when compared to normal tissues.

X-Ray Fluorescence (XRF) is a representative multielement technique for the analysis of trace elements [7,21,22,28,30-43]. This technique is based on exciting the atoms in a material by applying an X-ray beam with appropriate energy and subsequent detection of the characteristic radiation emitted, which in turn is proportional to the concentration of atoms in the material [44]. XRF has many advantages, such as a simple and rapid procedure of analysis in a large number of samples, high sensitivity and low detection limits, enabling the determination of elements concentrations in trace and ultra-trace levels [45,46]. The XRF technique has some variations depending on, among others, excitation and detection setup [46]. Basically, if excitation is performed under small angles relative to the sample, the technique is called Total Reflection X-Ray Fluorescence (TXRF), if incident beam is too small (order of microns), the technique is called Micro-X Ray Fluorescence (μ-XRF). If detection is performed in terms of wavelength or energy of radiation, the technique is called Wavelength Dispersive X-Ray Fluorescence (WDXRF) or Energy Dispersive X-Ray Fluorescence (EDXRF), respectively [46].

Therefore, the aim of this study was to evaluate the potential of trace elements as biomarkers and evaluate their prognostic value through EDXRF. To our knowledge, this is the first study to evaluate the potential of trace elements determined by X-ray fluorescence techniques as biomarkers and prognostic factors in breast cancer.

## Methods

### Patients

A total of 81 patients with normal, malignant and benign breast tissues diagnosed between 2003 and 2006 were randomly chosen from the files of the Department of Pathology of Ribeirão Preto Medical School in the University of São Paulo, at the Ribeirão Preto General Teaching Hospital (HCFMRP/USP). Clinical data and important prognostic information, such as age, menstrual status, tumor size, histological grade, staging, lymph node involvement, estrogen and progesterone receptors and HER-2 status were obtained from 59 available medical records. The study was approved by the local ethics committee (Research Ethics Committee of HCRP and FMRP-USP - Process 4308/2009) and followed the ethical guidelines of 1975 Helsinki Declaration.

### Breast tissue samples

Breast tissue samples were provided by the Department of Pathology at HCFMRP/USP. The collected material was residual tissue obtained from routine surgical procedures of mastectomy and mastoplasty. Pathological information was available for all samples. After surgery, samples were stored in formalin 10% until analysis. The patients were selected randomly to represent different aspects of breast tissues. The casuistic consisted of 38 samples of normal breast tissues (used as control group), 9 benign tumors (fibroadenoma) and 34 samples of malignant tumors (both in situ and infiltrating ductal carcinomas). In 25 of these 34 samples of malignant tumors, normal adjacent tissues were taken 2 cm away from tumors.

To perform XRF measurements, each sample was cut in 1 cm-thick and 1 cm-long sections, and placed in a specific sample holder, covered by Kapton® film, widely used in XRF experiments.

### X-ray fluorescence measurements

**Figure 1:**
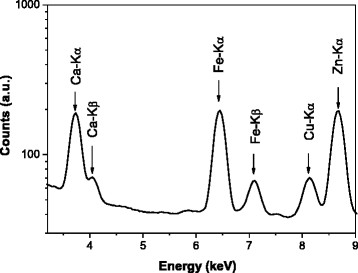
Typical EDXRF breast cancer spectrum.

Energy Dispersive X-Ray Fluorescence (EDXRF) experiments were performed at the Laboratory of Radiation Physics and Dosimetry, at the University of São Paulo (USP/Ribeirão Preto). A beam with energy of 17.44 keV emitted from an X-ray tube coupled with a graphite monochromator was used to excite simultaneously several trace elements in breast tissues. Samples were placed at a 45° angle to the incident beam in order to minimize attenuation effects [45]. For each sample, three datasets were collected from different regions of the sample to reduce the influence of a possible sample inhomogeneity. The acquisition time was fixed at 1000 s, maintaining the statistical uncertainty below 4%. Fluorescent signals emitted from trace elements were detected with a semiconductor detector Si(Li) (energy resolution of 165 eV at 5.89 keV), and placed at a 90° angle to the incident beam. Figure 1 shows a typical spectrum of EDXRF for a benign breast sample. One can observe the presence of several elements such as Ca, Fe, Cu and Zn. Trace elements concentrations were quantified by the external standard method associated with the scattered intensity method [7,45]. Detection limits were determined and the values range was between 0.2 mg.kg^-1^ (for heavier elements such as Zn) and 3.5 mg.kg^−1^ (for lighter elements such as Ca). A reference material (IAEA-V10) [47] with certified concentrations of several trace elements was used to test the method accuracy. Results revealed that differences between certified and obtained values were lower than 7% for all elements analyzed in the reference material, validating the quantification procedure.

### Receiver operating characteristics (ROC) analysis

In order to obtain decision threshold values (cut-off values) for each trace element, ROC curve analysis was employed [48]. The gold standard was based on histological diagnosis made by an experienced breast pathologist (ARS). ROC curves were generated based on concentration data of two types of tissues at a time. Table 1 summarizes the groups of tissues analyzed.

**Table 1:** Groups of tissues evaluated by ROC curve analysis

Group analyzed	Tissue established as disease present	Tissue established as disease absent
Group 1 (paired samples)	Malignant	Normal Adjacent
Group 2	Malignant	Normal
Group 3	Benign	Normal
Group 4	Benign	Malignant

The cut-off values for the concentration of trace elements chosen in this work corresponded to the point where the sensitivity and specificity were simultaneously higher [49,50]. Based on the cut-off value for each element, all trace elements concentration values were dichotomized in 0 or 1 (without or with tumor, respectively), transforming the distribution of the trace elements concentrations in a discrete variable (trace element expression).

### Evaluation of trace elements as tumor biomarker

The area under the ROC curve summarizes the performance of the test and the higher the discriminatory ability of the test, the more the area under the curve approaches to 1 [48]. In this work, the accuracy was considered excellent when the area under the curve was equal to or greater than 0.8 [26,50].

### Evaluation of trace elements as prognostic factors

Trace element expression (positive, 1, or negative, 0) was correlated with clinicopathological data using the Fisher’s exact test in the case of 2 variables and the chi-square test for three or more variables. The prognostic factors assessed were age, menstrual status, tumor size, histological grade, staging, lymph node involvement, estrogen and progesterone receptors, and also HER-2 status. The categorization of each prognostic factor was based on previous works described in the literature [51,52]. Correlation between trace elements and tumor size was performed only for malignant and benign breast tissues. Correlation between trace elements and histological grade, TNM staging, and lymph node status was conducted only for malignant tissues.

### Survival curves

The survival curves estimated by the Kaplan-Meier method were applied to study the isolated effect of trace element expression on the prognosis and to describe the cumulative proportions of deaths (overall survival) of patients [53]. These curves were built based on the expression of trace elements as independent variables and overall survival as the dependent variable. The time of diagnosis was considered the initial date and the event or failure was considered the occurrence of death. In this study, all cases of death were due to the presence of breast cancer. The cases that were not followed up until death were censored according to the Kaplan-Meier method [54]. The results were then compared using the log-rank test in order to determine statistically significant differences between the obtained curves. Statistical significance was based on confidence intervals of 95%.

The statistical analyses of data were performed using the Software SPSS® 13.0.

## Results and discussion

### Patients

In a total of 81 patients, 46.9% were diagnosed with normal breast tissue, 41.9% with malignant tumor and 11.2% with benign tumor. All patients were women, with a mean age of 50 years (range, 17 to 88 years of age). The mean size of the tumors was of 3.6 cm (range, 0.5 to 15 cm). Clinical features obtained from available medical records are reported in Table 2, according to tissue type.

**Table 2:** Clinicopathological characteristics of patients

	**Normal tissues**	**Malignant tissues**	**Benign tissues**
**Age (years)**			
≤ 30	2	0	4
30–50	14	14	5
50–70	2	9	1
≥ 70	1	8	0
**Menstrual status**			
Premenopausal	17	15	7
Postmenopausal	2	16	3
**Tumor size (cm)**			
≤ 2	−	4	1
2–5	−	20	7
≥ 5	−	7	1
**Histologic Grading**			
**(Bloom and Richardson)**			
G1	−	6	−
G 2	−	12	−
G 3	−	8	−
**Pathologic staging**			
I	−	0	−
II	−	10	−
III	−	9	−
IV	−	1	−
**Lymph nodes**			
Negative	−	11	−
1–3	–	4	–
≥ 3	–	15	–
**Death**			
Yes	0	10	0
No	19	21	9
**Estrogen receptor**			
Negative	–	12	–
Positive	–	21	–
**Progesterone receptor**			
Negative	–	13	–
Positive	–	20	–
**HER2**			
0	–	12	–
1+	–	7	–
2+	–	8	–
3+	–	6	–

### Trace elements concentrations

Trace elements concentrations data, determined by EDXRF, based on descriptive parameters (range, median, lower quartile, and upper quartile) are shown in Table 3. From this table it is possible to note that trace elements distributions are large, a common feature in biological parameters [55]. These variations in concentration may result from several factors, such as differences in diet, age, genetics and especially due to different disease stages [7,34,56]. Data in Table 3 also show that concentrations of trace elements are higher in neoplastic tissues (malignant and benign) than in normal tissues. Although the role of trace elements has not yet been well established in literature, the increase in tumor tissues may be related to metabolic and structural aspects [17,29,57-60], which indicates its potential as a tumor biomarker.

**Table 3:** Trace elements concentrations data in different tissues based on some descriptive parameters

**Element**	**Normal**	**Normal Adjacent**	**Malignant**	**Benign**
**Calcium**				
Mean	130.7	283.1	668.9	1770.3
Median	105.7	225.2	635.2	1320.3
Range	16.1–483.2	19.6–722.8	62.7–3572.5	300.9–4040.8
1^st^ and 3^rd^ quartile	68.9/186.6	154.5/342.0	271.0/793.8	795.5/2734.9
**Iron**				
Mean	15.6	15.8	33.0	15.1
Median	11.8	11.4	21.0	15.3
Range	3.6–87.9	4.9–103.4	8.7–166.9	5.5–26.6
1^st^ and 3^rd^ quartile	6.4/20.2	7.3/16.0	12.8/42.6	11.2/18.7
**Copper**				
Mean	3.3	0.6	1.0	2.1
Median	0.4	0.5	0.7	1.6
Range	0.1–103.0	0.1–1.7	0.2–5.4	0.9–6.7
1^st^ and 3^rd^ quartile	0.2/0.7	0.3/0.9	0.5/1.3	1.7/1.9
**Zinc**				
Mean	2.1	2.8	7.1	12.2
Median	1.5	2.3	6.6	11.1
Range	0.1–9.7	0.3–8.8	1.6–30.0	4.5–21.1
1^st^ and 3^rd^ quartile	0.4/3.2	1.7/3.5	3.9/8.4	6.9/15.5

First and third quartiles correspond to the 25th and 75th percentiles respectively. All values are in mg/kg.

Table 3 also shows that, except for Fe, all elements concentrations are higher in benign tumors than in malignant tumors. This finding is in agreement with other authors [7,21,27,30,33,34] and may be associated with structural [59,61,62] and metabolic aspects [18,19,63-67] as well as tumor development [68,69].

### Cut-off determination

**Figure 2:**
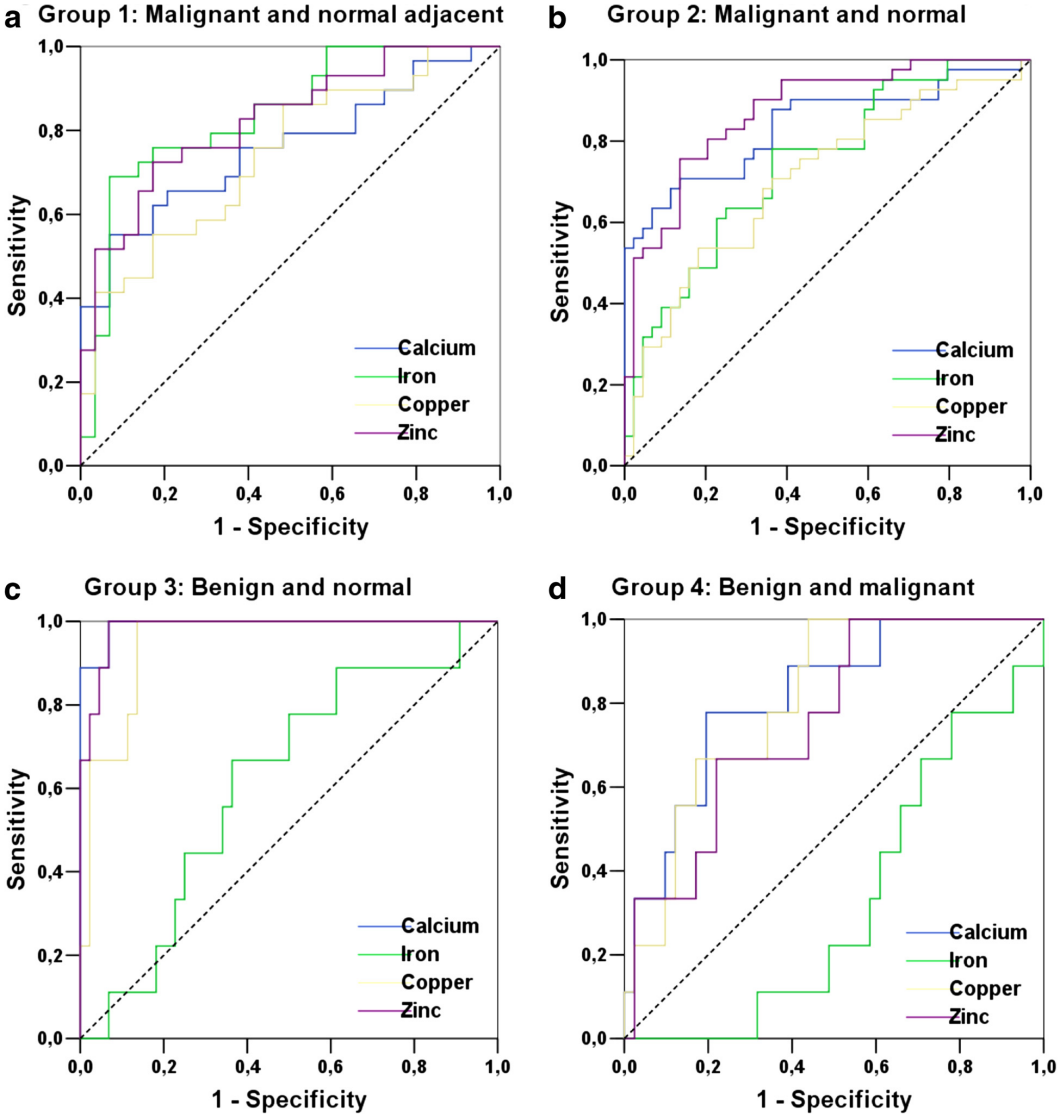
**ROC curves obtained from the study of trace element concentration distributions.** Group 1: malignant and normal adjacent, group 2: malignant and normal, group 3: benign and normal, group 4: benign and malignant. Reference lines are shown in dotted lines.

Figure 2 shows the ROC curves obtained for all elements studied in this work, comparing different types of tissues. In the same way that areas under the ROC curves provided a measure of accuracy of the test (diagnostic), the coordinates of sensitivity and specificity are also important because they provide information to assist in the decision of the cut-off value used to determine the outcome of the trial. Table 4 describes the sensitivity and specificity associated with the cut-off value chosen, as well as the values of the areas under the curves and the asymptotic significance in area determination. The cut-off limits were selected based on the highest sum of sensitivity and specificity [49].

**Table 4:** ROC graphs parameters obtained for trace elements present in different tissues

Group 1: Malignant (1) and normal adjacent tissues (0)-paired samples					
Element	Best cut-off (mg.kg^-1^)	Sensitivity (%)	Specificity (%)	Area under ROC curve	p
Ca	580.9	55.2	93.1	0.757	0.001
Fe*	**19.3**	**69.0**	**93.1**	**0.837**	**<0.001**
Cu	1.0	55.2	82.8	0.740	0.002
Zn*	**4.0**	**72.4**	**82.8**	**0.824**	**<0.001**
Group 2: Malignant (1) and normal tissues (0)					
Ca*	**212.6**	**70.7**	**86.4**	**0.838**	**<0.001**
Fe	12.6	78.0	63.6	0.743	<0.001
Cu	0.8	53.7	81.8	0.707	0.001
Zn*	**3.4**	**75.6**	**86.4**	**0.874**	**<0.001**
Group 3: Benign (1) and normal tissues (0)					
Ca*	**288.8**	**100.0**	**93.2**	**0.992**	**<0.001**
Fe	12.8	66.7	63.6	0.616	0.276
Cu*	**0.9**	**100.0**	**86.3**	**0.947**	**<0.001**
Zn*	**4.4**	**100.0**	**93.1**	**0.985**	**<0.001**
Group 4: Benign (1) and malignant tissues (0)/^Δ^ Benign (0) and malignant tissues(1)					
Ca*	**794.6**	**77.8**	**80.5**	**0.816**	**0.003**
Fe ^Δ^	20.7	48.7	88.8	0.675	0.103
Cu*	**0.9**	**100.0**	**56.1**	**0.808**	**0.004**
Zn	4.4	100.0	46.3	0.759	0.016

The values 1 and 0 used in graphs construction represent tissues considered with presence and absence of disease, respectively. Elements highlight by * show area under the ROC curve higher than 0.8.

### Trace elements as tumor biomarkers and prognostic factors

**Figure 3:**
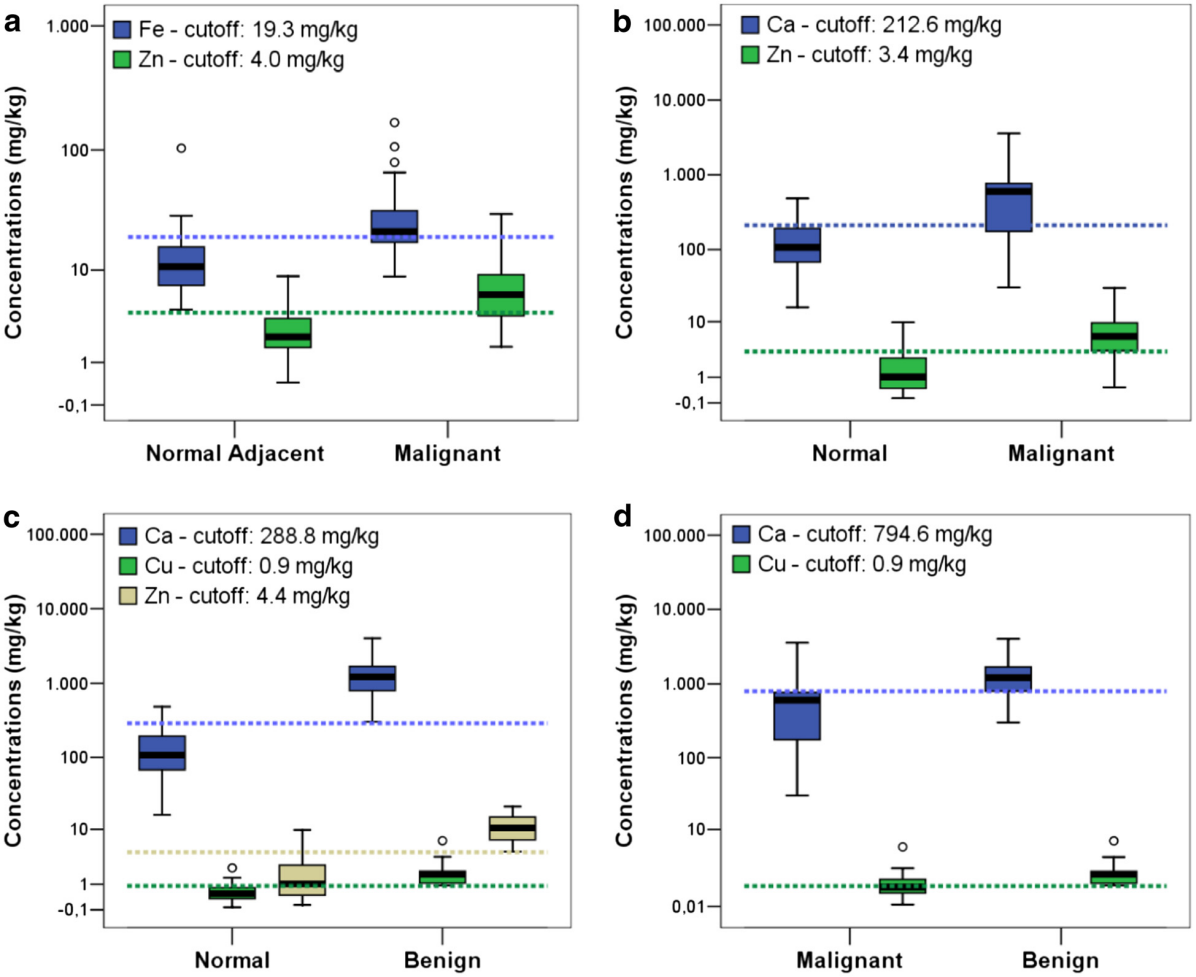
**Box plots of trace elements in different breast tissues.** (**a**) normal adjacent and malignant (**b**) normal and malignant (**c**) normal and benign and (**d**) malignant and benign. The circles indicate outliers, which are values between 1.5 and 3 times the inter quartile ranges. Color dotted lines represent the cut-off values for each correspondent color distribution.

From Table 4 it is possible to observe that for each test, some elements (highlight by *) show the area under the ROC curve higher than 0.8. Concentrations distribution of these elements and respective cut-off values are shown in Figure 3 through box plot graphs, which makes it possible to represent the distribution of concentrations of trace elements based on descriptive parameters such as range, median and quartile range [70]. From Figure 3 (a) it is possible to identify that the cut-off values for the elements Fe and Zn are used to classify normal adjacent and malignant tissues with high sensitivity and specificity (values on Table 4). Figures 3(b)3(c) and 3(d) illustrate the distributions of concentrations and their respective cut-off values for other trace elements which made it possible to classify other groups of breast tissues. It is also observed in Table 4 that all trace elements may be considered as tumor biomarkers once they show areas under ROC curve near 0.8. However, these elements could distinguish breast tumors with a lower accuracy.

The assigned value to each variable (0 or 1), based on the cut-off value, allowed us to assess the expression of trace elements, and correlate them with clinicopathological data, with the results shown in Table 5. In this table, it is possible to note that the expression of all trace elements is correlated with the type of tissue. These findings emphasize that there is a possibility of using these elements as tumor biomarkers.

**Table 5:** **Correlation between trace elements expression, tissue type and** prognostic factors

	**Calcium status**		**Iron status**		**Copper status**		**Zinc status**	
	**−**	**+**	**−**	**+**	**−**	**+**	**−**	**+**
**Breast Tissue**								
Normal	33	5	22	16	29	9	31	7
Malignant	7	27	6	28	17	17	7	27
Benign	0	9	3	6	0	9	0	9
Normal Adjacent	11	14	13	12	16	9	17	8
*p*	<0.001		0.005		<0.001		<0.001	
**Age (years)**								
< 30	0	6	4	2	0	6	0	6
30–50	17	16	11	22	22	11	17	16
50–70	3	9	2	10	6	6	4	8
> 70	1	8	0	9	4	5	0	9
*p*	0.019		0.028		0.023		0.007	
**Menstrual status**								
Premenopausal	17	22	17	22	26	13	17	22
Postmenopausal	4	17	0	21	6	15	4	17
*p*	0.050		<0.001		0.005		0.05	
**Death**								
Yes	17	33	17	33	30	20	19	31
No	4	6	0	10	2	8	2	8
*p*	0.490		0.025		0.024		0.239	
**Tumor size (cm)**								
≤ 2	1	4	1	4	2	3	1	4
2–5	5	22	5	22	10	17	5	22
≤ 5	1	7	2	6	4	4	1	7
*p*	0.914		0.922		0.806		0.914	
**Histologic Grading (Bloom and Richardson)**								
G1	1	5	1	5	5	1	2	4
G 2	1	11	0	12	5	7	1	11
G 3	3	5	2	6	5	3	4	4
p	0.264		0.208		0.228		0.111	
**Pathologic staging**								
II	4	6	1	9	3	7	4	6
III	3	6	2	7	3	6	2	7
IV	0	1	0	1	0	1	1	0
*p*	0.719		0.69		0.788		0.271	
**Lymph nodes**								
Negative	2	9	2	9	8	3	2	9
1–3	0	4	0	4	2	2	0	4
≥ 3	5	10	3	12	6	9	5	10
*p*	0.330		0.626		0.253		0.33	
**Estrogen receptor**								
Negative	3	9	2	10	6	6	2	10
Positive	5	16	3	18	12	9	6	15
*p*	0.627		0.612		0.486		0.373	
**Progesterone receptor**								
Negative	2	11	2	11	7	6	2	11
Positive	6	14	3	17	11	9	6	14
*p*	0.299		0.669		0.614		0.299	
**HER2**								
score 0	3	9	3	9	10	2	5	7
score 1+	2	5	0	7	3	4	1	6
score 2+	1	7	0	8	2	6	1	7
score 3+	2	4	2	4	3	3	1	5
*p*	0.814		0.163		0.064		0.369	

(−) and (+) represents negative and positive expression respectively. The values of significance for Fisher and chi-square tests are represented by p.

Table 5 also shows that the trace elements expression is statistically correlated with age and menstrual status. Regarding the menstrual status of the patients, our results indicate that most post-menopause women have a positive correlation with the expression of trace elements. There were no statistically significant correlations for the other factors evaluated. However, the number of patients in each group of prognostic factors may have been too small to detect a statistically significant correlation. Furthermore, since prognostic factors evaluated in Table 5 are independent [1,2,51,71-75], the correlation with the tissue type, age and menopausal status suggests that expression of trace elements may be considered a prognostic factor.

**Figure 4:**
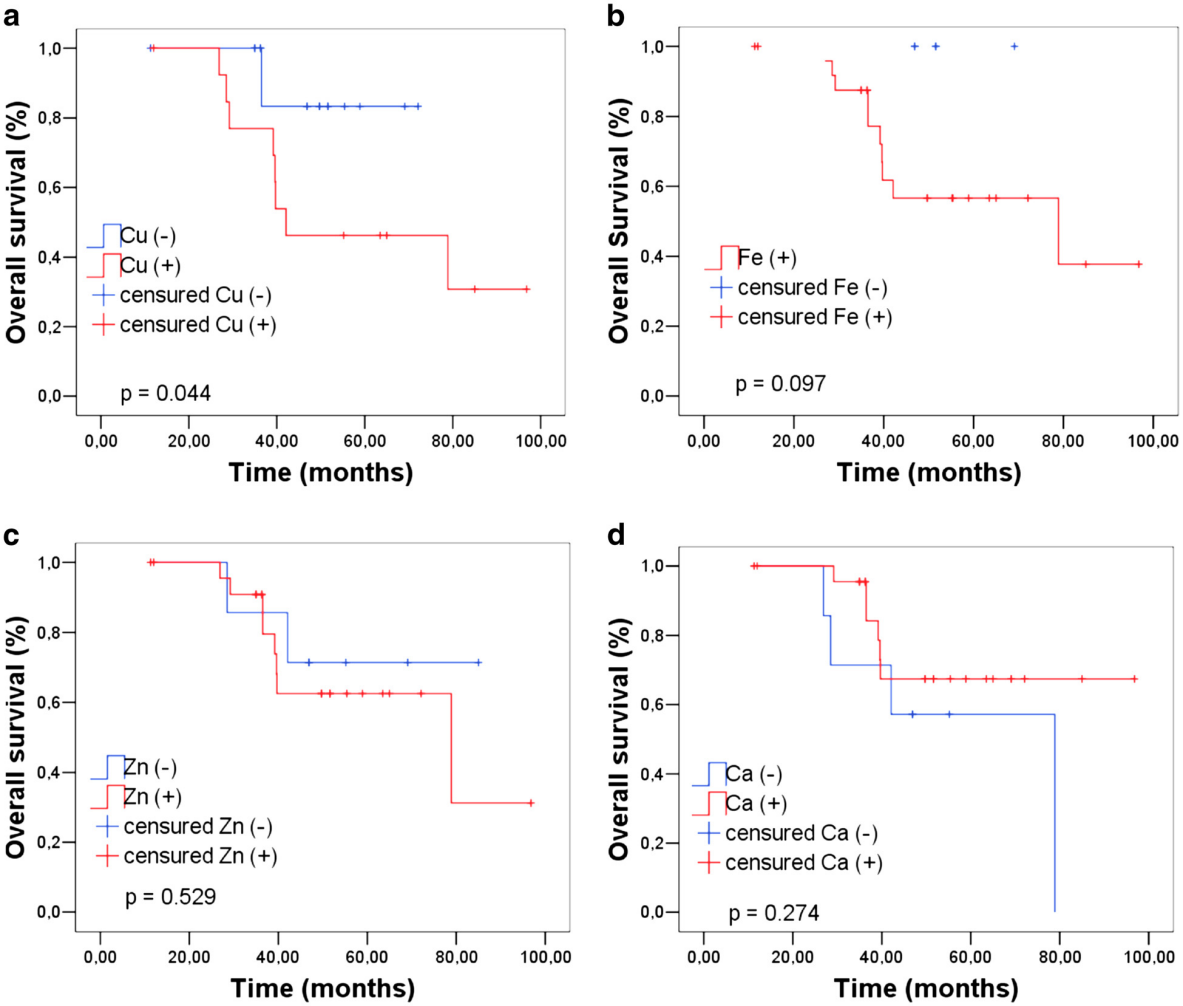
Overall survival curves as a function of trace elements expression.

Survival curves estimated by the Kaplan-Meier method, used to examine the expression of trace elements as a function of overall survival of patients diagnosed with malignant tumors, and the values of the log-rank tests are presented in Figure 4. Figure 4 (a) shows that patients with positive expression for Cu presented a poor overall survival (*p* < 0.001). Indeed, studies report that copper has a crucial role in the angiogenic mechanism, and tumors that become angiogenic exhibit a high metastatic potential, a major cause of mortality in patients with breast cancer [18,76]. In addition, in order to confirm this hypothesis, it would be interesting in future studies to examine the presence of angiogenic vessels in these cases.

Even without showing statistically significant difference for the other elements, the graphs in Figures 4(b) and 4(c) indicate that the positive expression for Fe and Zn is associated with an increased risk of death. Data in Figure 4b show that there were no deaths among patients with negative expression for Fe, thus the overall survival did not need to be recalculated at any time and only censored data are presented.

Calcium (Figure 4 (d)) presents an opposite trend. This opposite association of calcium with poor survival can be associated with the presence of microcalcifications in tissue, once differences in the structures of microcalcifications may be inversely associated with the degree of malignancy [14,61,62].

The results of this work revealed that the concentrations of trace elements, determined by EDXRF, are important objects of study to be established as prognostic factors in the near future, since the larger the number of biomarkers, the higher the number of possible combinations that can help the physician to establish the appropriate therapy for each case, providing a more individualized therapeutic management and improving the selection of patients for adjuvant therapy.

## Conclusions

In this work, Energy Dispersive X-Ray Fluorescence technique was employed to determine Ca, Fe, Cu, and Zn concentrations in normal and neoplastic breast tissues. Cut-off values, obtained by ROC analysis, allowed us to distinguish different types of breast tissues, including malignant and benign. In addition, the correlations between trace elements expression and tissue type suggest that trace elements can be used as tumor biomarkers.

The prognostic value of the trace elements was demonstrated through the correlation between their expression and parameters such as age and menstrual status. Moreover, patients with positive copper expression showed a poorer survival. The greater the number of prognostic factors, the greater the number of possible combinations that can help doctors identify patients with very aggressive tumors and institute appropriate therapy for each case. In addition, doctors may also consider the effectiveness of different treatment alternatives and the cost-benefit to the patient.

The results in this work suggest that the expression of trace elements is a clinically relevant potential tool that could be integrated in decision-making, making it possible to individualize the treatment and to develop target therapies based on trace elements expression.

## Misc

Marina P Silva, Danilo F Soave, Alfredo Ribeiro-Silva and Martin E Poletti contributed equally to this work

## Authors’ contributions

MEP coordinated the x-ray fluorescence measurements and the study design. MPS conducted the sample collection, the x-ray fluorescence measurements, the computations and the statistical analysis, the collection of patients’ clinical data and the manuscript drafting. DFS contributed with collection of patients’ clinical data. ARS contributed with study design, analysis and data interpretation. All authors read and approved the final manuscript.

## Competing interests

The authors declare that they have no competing interests.
